# Role and significance of SIRT1 in regulating the LPS-activated pyroptosis pathway in children with congenital hydronephrosis

**DOI:** 10.1136/wjps-2023-000602

**Published:** 2023-07-31

**Authors:** Zhan Wang, Gu Weizhong, Juan Zhou, Daxing Tang

**Affiliations:** 1 Department of Urological surgery, Children's Hospital, Zhejiang University School of Medicine, National Clinical Research Center for Child Health, Hangzhou, China; 2 Department of Pathology, Children's Hospital, Zhejiang University School of Medicine, National Clinical Research Center for Child Health, Hangzhou, China

**Keywords:** child health

## Abstract

**Objective:**

To explore the characteristics and mechanism of sirtuin 1 (SIRT1) in lipopolysaccharide (LPS)-activated pyroptosis in the renal tissue of children with congenital hydronephrosis (CHn).

**Methods:**

We detected the expression characteristics and clinical significance of SIRT1 and pyroptosis pathway proteins in CHn renal tissues by immunohistochemistry. The degree of renal fibrosis was detected by Masson staining. The human renal tubular epithelial cell line (HK-2) was cultured in vitro and treated with LPS (1 µg/mL), the SIRT1-specific agonist SRT1720 (2.5 µmol/L) and small interfering RNA (siRNA)-SIRT1 for 48 hours. After 48 hours, Cell Counting Kit-8 was used to detect the changes in cell proliferation ability, and ELISA was used to detect the changes in the expression of interleukin (IL)-1β and IL-18 in the cell supernatant. Real-time PCR (quantitative RT-PCR) and western blot analysis were used to detect the expression of SIRT1, caspase-1, caspase-4, NOD-like receptor thermal protein domain associated protein 3(NLRP3), and cleaved gasdermin D (GSDMD) in each group.

**Results:**

Serum inflammatory cytokines were significantly elevated in 13 children with CHn with urinary tract infection, mainly caused by Gram-negative bacteria. Severe renal fibrosis occurred in children with CHn. Compared with the control group, the expression of SIRT1 in CHn kidney tissues was decreased, and the expression of caspase-4 and GSDMD was increased. LPS inhibited the expression of SIRT1 in HK-2 cells, promoted the expression of caspase-1, caspase-4, NLRP3, cleaved GSDMD, promoted the expression of IL-1β and IL-18 in the supernatant, and promoted pyroptosis in HK-2 cells. SRT1720 can inhibit LPS-activated pyroptosis by promoting SIRT1 expression, while siRNA-SIRT1 can further aggravate LPS-activated pyroptosis after inhibiting SIRT1 expression.

**Conclusions:**

LPS can promote the inflammatory response in children with CHn by activating non-canonical pyroptosis and inhibiting SIRT1 expression. Promoting SIRT1 expression can inhibit pyroptosis of renal tubular epithelial cells, reduce the release of IL-18 and IL-1β, and alleviate the progression of renal fibrosis in children with CHn.

**WHAT IS ALREADY KNOWN ON THIS TOPIC**

- The search for molecular markers or targets for the diagnosis and treatment of congenital hydronephrosis (CHn) is a hot topic for researchers.
- It has been confirmed that the canonical pyroptosis pathway can promote the progression of renal interstitial fibrosis in renal disease.
- At present, the role and mechanism of the non-canonical pyroptosis pathway in CHn is unclear.
- This study aims to explore the role of lipopolysaccharide (LPS)-activated non-canonical pyroptosis pathway in CHn and to find targets for improving renal interstitial fibrosis in CHn.

**WHAT THIS STUDY ADDS**

- In this study, it was found that LPS can activate the non-canonical pyroptosis pathway mediated by caspase-4 in CHn, which may be an important mechanism of LPS promoting the progression of renal interstitial fibrosis in CHn.
- Sirtuin 1 (SIRT1) can inhibit pyroptosis in CHn and reduce the release of inflammatory cytokines interleukin (IL)-1β and IL-18, which may be a target for relieving renal interstitial fibrosis of CHn.

**HOW THIS STUDY MIGHT AFFECT RESEARCH, PRACTICE OR POLICY**

- The results of this study suggest that SIRT1 can regulate the progression of renal interstitial fibrosis in CHn by inhibiting the non-canonical pyroptosis pathway.
- Promoting SIRT1 expression and inhibiting caspase-4-mediated non-canonical pyroptosis pathway can alleviate the progression of renal interstitial fibrosis in CHn.
- On the basis of this study, this conclusion can be further confirmed by animal experiments and clinical studies in the future.
- The results could help researchers search for new molecular markers to treat CHn.

## Introduction

Congenital hydronephrosis (CHn) is a common disease in pediatric urology, and its most common cause is ureteropelvic junction obstruction (UPJO). The disease may lead to high pelvic pressure, thus putting pressure on the renal parenchyma and causing damage to renal function. Children suffering from hydronephrosis are prone to urinary infection that triggers more damage, which can lead to severe renal fibrosis and renal dysplasia unless they are treated promptly and effectively. The inflammatory response activated by cell wall components of Gram-negative bacterial lipopolysaccharide (LPS) is an important mechanism promoting the progression of renal interstitial fibrosis in children with CHn.^1^ The mechanism may be that inflammation promotes the infiltration of macrophages and secretes fibrosis-promoting factors such as transforming growth factor (TGF)-β1, basic fibroblast generating factor, and interleukin (IL)-1 to aggravate renal interstitial fibrosis.^2^ More inflammatory factors induce excessive apoptosis of renal tubular epithelial cells (RTECs) and weaken the ability of tissue repair. Inflammation also promotes epithelial interstitial transformation (EMT) of RTECs into fibroblasts, producing more collagenous fibers and leading to the progression of renal interstitial fibrosis.^3^ For this reason, it is important to find some effective measures to reduce the inflammatory reaction in the renal tissue of children with CHn. These measures can slow the progression of renal fibrosis, save renal function to a certain extent and have positive significance for clinical comprehensive treatment of children with CHn.

Pyroptosis is a newly discovered pattern of programmed cell death that has been a research hotspot in recent years for infectious diseases and autoimmune diseases. Pyroptosis may cause rapid rupture of the cytoplasmic membrane and release of cell contents, including some pro-inflammatory factors, such as IL-1β and IL-18, which produce strong inflammatory reactions in surrounding cells and tissues.^4^ There are two main pathways of pyroptosis: the NLRP3/caspase-1-mediated canonical pathway and the caspase-4/5/11-mediated non-canonical pathway.^5^ Gasdermin D (GSDMD), a member of the gasdermin family, is the co-substrate of caspase-1/4/5/11 and is the executive protein of pyroptosis.^6^ A study found that in acute kidney injury^7^ and diabetic kidney disease (DKD) models, pyroptosis was activated to increase inflammatory reactions and tissue damage, and the inhibition of pyroptosis alleviated inflammatory damage.^8 9^ Inflammatory reactions and cell injury due to pyroptosis were closely related to the progression of DKD, exacerbation of renal fibrosis, glomerular sclerosis and renal tubular damage.^10^ GSDMD inhibitors can inhibit the pyroptosis of human renal tubular epithelial cells (HK-2) activated by LPS, reduce the release of inflammatory cytokines, and thus reduce the expression of profibrotic cytokines.^11^ Based on this, we hypothesized that the mechanism by which LPS promotes the progression of renal interstitial fibrosis in CHn may be that LPS promotes inflammation by activating HK-2 to induce pyroptosis and then promotes renal interstitial fibrosis.

In studies of renal diseases, sirtuin 1 (SIRT1) can inhibit the expression of various inflammatory signaling pathways to regulate renal interstitial fibrosis.^12^ It has been found that overexpression of SIRT1 can inhibit inflammatory reactions by inhibiting the NLRP3/caspase-1-mediated canonical pyroptosis pathway and protect cells and tissues.^13 14^ LPS can promote inflammation by inhibiting the expression of SIRT1 in cells, and agonists of SIRT1 can reduce the cell damage and inflammatory response induced by LPS. SIRT1 is the target of LPS.^15^ However, the role of SIRT1 in LPS-activated pyroptosis, especially in non-classical pyroptosis, and the mechanism of this pathway in CHn renal interstitial fibrosis remain unclear. Therefore, this study intends to explore the role of SIRT1 in LPS-activated pyroptosis through clinical specimen experiments and in vitro cell experiments, hoping to help find targets or drugs for the treatment of renal interstitial fibrosis in children with CHn.

## Materials and methods

### Subjects and specimen collection

Thirteen children with CHn who underwent nephrectomy from January 2013 to December 2021 at our urology surgery were selected as subjects in the study. Inclusion criteria are as follows: (1) UPJO is the cause of CHn and its postoperative pathology diagnosis; (2) indications for nephrectomy are clear. On the premise of good contralateral kidney function, the indications of nephrectomy in severe hydronephrosis kidney should meet either of the following conditions: (1) diuretic renal nuclide imaging indicates that the kidney has no function or function is <5%–10%, and renal function is still <5%–10% in re-examination 4~12 weeks after nephrostomy; (2) the diuretic renal nuclide imaging has no function or <5%~10% function, and the renal parenchyma is thin, pale and tough after releasing urine during the operation; (3) diuretic renal radionuclide imaging suggested renal non-function and pyonephrosis.^16^ Exclusion criteria contain renal failures caused by congenital renal dysplasia, polycystic kidney and other causes. Kidney tissue specimens from 13 children with CHn were defined as the observation group, and paracancerous tissue specimens from 5 children with nephroblastoma were used as the control group (>2 cm away from the tumor edge).

The medical history and clinical data of 13 children with CHn were collected, including high-sensitivity C reactive protein (hs-CRP) (reference range 0–8 mg/mL), Procalcitonin (0.0–0.460 ng/mL), IL-6 (1.7–16.6 pg/mL), tumor necrosis factor (TNF) (0.1–5.2 pg/mL) and interferon (IFN)-γ (1.6–17.3 pg/mL), urine specimen bacterial culture, test results and other clinical data (the results of bacterial identification in our hospital only showed dominant colony growth).

### Experimental methods

#### Cells and major reagents

Human renal tubular epithelial cell line (HK-2) was employed as the cell strain (obtained from the cell bank of American Type Culture Collection). Primary antibodies were: mouse antihuman SIRT1 antibody (Abcam, USA, ab110304); rabbit antihuman caspase-1 antibody (22915-1-AP, Proteintech, USA); rabbit antihuman NLRP3 antibody (19771-1-AP, Proteintech, USA); rabbit antihuman caspase-4 polyclonal antibody (ab25898, USA); rabbit antihuman cleaved GSDMD (Asp275) (E7H9G) rabbit mAb (#36425, Cell Signaling Technology, USA); Cell Counting Kit-8 (CCK8) Cell Proliferation Kit (DOJINDO, Japan); human IL-1β and IL-18 ELISA Kit (Elabscience, Wuhan, China); LPS (L6143, Sigma, USA) and SRT1720 (catalog no. A4180, APExBIO, USA). Goat antirabbit secondary antibody, small interfering RNA (siRNA)-SIRT1 gene sequence and quantitative RT (qRT)-PCR-related reagents were obtained from Zhongyan Biological (Beijing, China).

#### Immunohistochemical staining

All specimens were fixed with 10% neutral buffer—formalin for storage and then conventionally dehydrated, paraffin-embedded tissue was made into tissue blocks. The collected paraffin tissue blocks were continuously sliced, approximately 4 µm thick, and 6 pieces of each specimen were cut for immunohistochemical (IHC) and Masson staining. All operations involving IHC staining were performed by Leica Bond 3 (Germany), an automatic IHC staining machine. The working concentrations of primary antibodies against SIRT1 and caspase-1 were 1:150, NLRP3 was 1:100, caspase-4 was 2 µg/mL, and cleaved GSDMD was 1:500.

#### Immunohistochemical staining results

Three non-overlapping fields of view were randomly selected at 200× magnification using CaseViewer. The area of view was the renal parenchyma, with each visual field covering glomeruli and tubules. Positive SIRT1 expression was detected in the nucleus, and the caspase-1, NLRP3, and caspase-4 proteins were mostly detected in the cytoplasm and nucleus, which is characterized by clear yellow and brown granules. The positive expression of cleaved GSDMD protein was mainly found in the cell membrane that presented clear brown or brown granules. Image-Pro plus 6.0 was adopted to analyze the images and calculate the mean optical density (MOD) for each, MOD=integral optical density/total area (kidney tissue).

#### CCK8 proliferation assay of HK-2 cells

The cells were digested and resuspended in complete medium, counted and then inoculated into 96-well plate, with 35 000 cells per well. After 18 hours, LPS was added to the plate, and culture media containing different final concentrations of LPS (0.0, 0.5, 1.0, 5.0, 10.0 µg/mL) were prepared with basic medium. After LPS treatment for 48 hours, the culture media were removed and replaced with complete medium containing 10% CCK8 per well, as a blank group, with CCK8 and medium alone and without cells, then another 1 hour of culture was carried out. One hour later, the light absorption value at 450 nm was measured on a microplate reader. Calculation formula: cell viability (%)=(optical density (OD) of experimental group−OD of the blank control group)/(OD of negative control group−OD of the blank control group)×100. Based on the experimental results, the final concentration of LPS was 1.0 µg/mL for building the model in the follow-up experiment, and equally, the impacts of SRT1720 and siRNA-SIRT1 on the proliferation of HK-2 cells were detected.

#### ELISA detection of IL-1β and IL-18 in the supernatant of HK-2 cells

The cells were digested and resuspended in complete medium, counted and then inoculated into 6-well plate, at 330 000 cells per well. After 18 hours, different groups were set up, and drugs were given to intervene in the cells. LPS was diluted with Dulbecco’s Modified Eagle Medium-based medium. The dosage of culture medium for each group was changed to 1.5 mL. Forty-eight hours after administration, the supernatant was collected and centrifuged for 20 min at a centrifugal force of 1000 g to remove impurities and cell debris, and the supernatant was collected for ELISA. The concentrations of IL-1β and IL-18 in the supernatant were measured by following the instructions of the ELISA kit.

#### siRNA-SIRT1 experiment and SRT1720 concentration screening of HK-2 cells

Three siRNA-SIRT1 gene sequences were constructed and detected (online supplemental table 1). A blank control group and a siRNA-negative control group were established. HK-2 cells were inoculated into 6-well cell culture plate and transfected when the cell density was approximately 70%. The cells were transfected as per the operation process of siRNA. Forty-eight hours later, the total RNA from the cells of each group was extracted for qRT-PCR detection. The gene sequence with the highest inhibition rate was screened for subsequent experiments. The final concentration of SRT1720 was set to 0.0 µmol/L, 1.25 µmol/L, 2.5 µmol/L, and 5.0 µmol/L. HK-2 cells were inoculated into 6-well plate, and SRT1720 was added when the cell density reached approximately 70%. After 48 hours, the total RNA of each group was extracted for qRT-PCR detection. A suitable final concentration was screened for subsequent experiments.

10.1136/wjps-2023-000602.supp1 Supplementary data

#### Real-time PCR (qRT-PCR) test of HK-2 cells

Total RNA was extracted from each group according to the kit for qRT-PCR detection. Real-time PCR (qRT-PCR) was performed using SYBR (Promega) according to the instrument manual (HT7500 System; Applied Biosystems, Foster, USA). Relative expression was calculated using the 2^−△△Ct^ method. GAPDH was amplified as a control for RNA integrity. The sequences of the primers and reaction systems are listed in online supplemental table 2.

#### Western blot analysis of HK-2 cells

HK-2 cells were inoculated into a 6-well plate at 330 000 cells per well. After 18 hours, group-based interventions were performed: blank control group, LPS group, LPS+SRT1720 group, LPS+dimethylsulfoxide group, LPS+siRNA-SIRT1 group, and LPS+siRNA Negative control (NC) group. Following 48 hours of incubation, the cell proteins of each group were collected for western blot analysis. Under the instructions of the Protein Extraction Kit (strong), protein was extracted from each group of cells. The protein concentration was adjusted by water and sodium dodecyl sulfate-polyacrylamide gel electrophoresis (SDS-PAGE) and boiled at 100℃ for 10 min. According to SDS-PAGE specifications, a 12% separating gel and 5% stacking gel were prepared based on the molecular weight of the target protein. The protein sample was loaded at 50 µg, transferred to a polyvinylidene difluoride membrane, blocked with 5% skimmed milk powder for 1 hour, diluted with 1% bovine serum albumin/5% milk (the working concentration of SIRT1 was 1:3000, and those of caspase-1, caspase-4, NLRP3 and cleaved GSDMD were 1:1000), and incubated overnight at 4℃. Dilute two antibodies with 5% skimmed milk powder, sheep antirabbit-HRP or sheep antimice anti-HRP (choose the corresponding two antibodies according to the source of one antibody) 1∶5000 dilution at room temperature for 50 min. The membrane was washed before adding Enhanced chemiluminescence and exposing plate, and the gray value of the strip was measured by ImageJ.

### Statistical method

The data analysis was conducted using SPSS V.23.0 and GraphPad Prism V.7.0 software. Following the normal distribution test, the qualified quantitative data were expressed as the mean±standard deviation (SD) and compared with the χ^2^ test and unpaired t-test. Unqualified data were expressed as M(Q1, Q3), and the Mann-Whitney U test was employed. P values of <0.05 indicated statistically significant differences.

## Results

### Summarized clinical data of 13 children with severe hydronephrosis

The 13 children with CHn included 8 males and 5 females, of whom the youngest was 4 months and 6 days and the oldest was approximately 14 years 6 months. In 13 children with CHn, urinary tract infections occurred once or more in the past course of disease before nephrectomy. The hs-CRP in the serum of children with urinary tract infection was 98.21±19.27 mg/mL, and the values of PCT, IL-6, TNF, and IFN-γ were 4.713±1.488 ng/mL, 422.7±160.0 pg/mL, 1.908±0.1966 pg/mL, and 37.22±15.05 pg/mL, respectively. The results of urine culture and colony count in the 13 children demonstrated that there were 6 *Escherichia coli* infections, 2 *Pseudomonas aeruginosa* infections, 2 *Klebsiella pneumoniae* infections and 10 Gram-negative bacterial infections. These clinical findings suggest that children with CHn are more likely to develop urinary tract infections caused by Gram-negative bacteria, along with overexpression of pro-inflammatory cytokines.

### Pathological features of renal tissue in children with severe hydronephrosis

**Figure 1:**
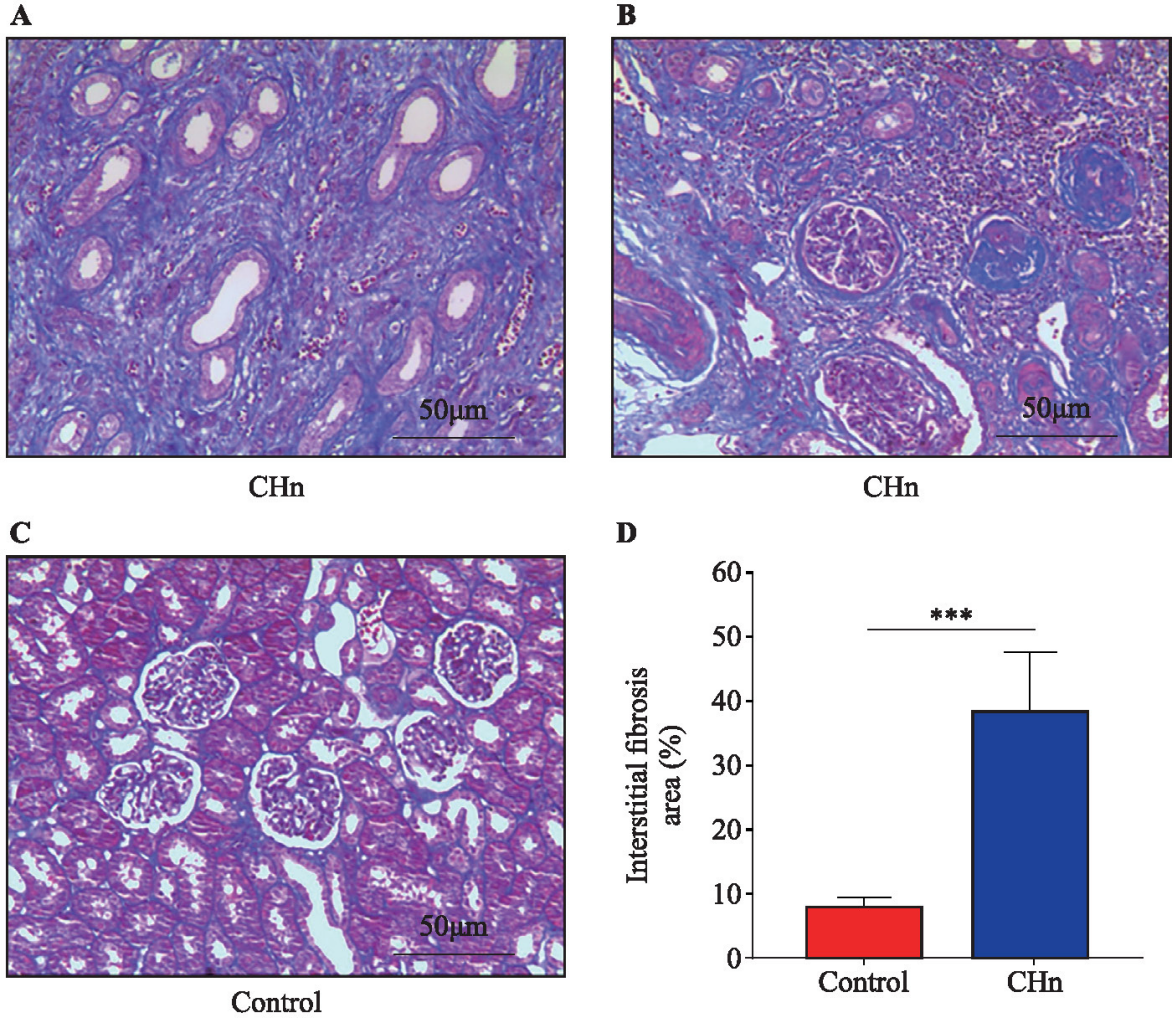
The Masson staining of renal tissue in both groups. (A and B) Severe interstitial fibrosis, reduced number of renal tubules and atrophy, reduced number of glomeruli, and glomerular fibrosis were observed in the kidney tissue on the affected side of congenital hydronephrosis (CHn). (C) In the control group, there was no obvious fibrosis in renal tissue, and the morphology of renal tubules and glomeruli was normal. (D) The comparison of renal interstitial fibrosis area between the two groups. (***p<0.001, compared with control group, 5 cases in the control group and 13 cases in the CHn group).

Masson staining of renal tissue showed renal cortical atrophy, evident proliferation of fibrous tissue in the renal parenchyma, tubular and glomerular compression, structural destruction, and glomerulosclerosis in children with CHn (figure 1). The area of interstitial fibrosis in the CHn group was 38.53% (30.92%, 46.06%), which was markedly higher than that in the control group (9.83%, 6.11%, 11.07%).

### Expression of SIRT1 and pyroptosis pathway proteins in renal tissues of children with CHn

**Figure 2:**
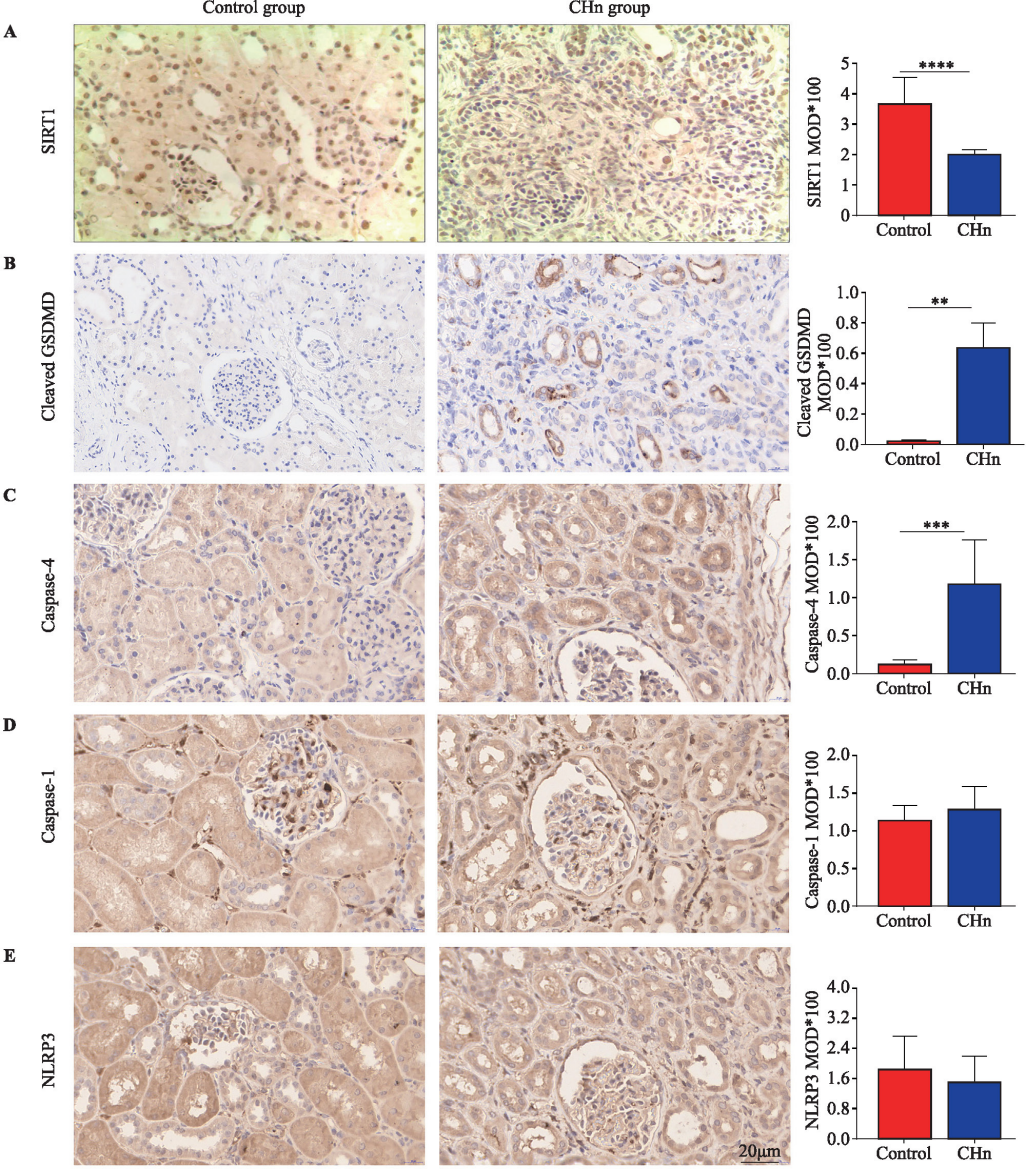
Expression of sirtuin 1 (SIRT1) and pyroptosis pathway proteins in renal tissues of both groups. (A) The expression of SIRT1 protein in renal tissues of the two groups was decreased in congenital hydronephrosis (CHn). (B) The cleaved gasdermin D (GSDMD) protein was overexpressed in CHn group and merely expressed on the cell membrane of renal tubular epithelial cells. (C) The expression of caspase-4 protein in renal tissues of the two groups was increased in CHn. (D) The caspase-1 p20 protein was widely expressed in kidney tissue, showing no statistical difference between both groups. (E) The activity NLRP3 protein was widely expressed in renal tissue, showing no statistical difference between both groups (**p<0.01, ***p<0.001, ****p<0.0001 compared with control group, 5 cases in the control group and 13 cases in the CHn group).

SIRT1 is widely expressed in normal kidney tissues, mainly in the nucleus. Compared with the control group, the expression level of SIRT1 in CHn kidney tissues was decreased (figure 2A). Activated caspase-1/4/5/11 can cleave GSDMD to form GSDMD-N and GSDMD-C terminals. The GSDMD-N-terminus is the main functional domain that can target the cell membrane, bind to the phospholipoprotein on the cell membrane, polymerize and form pores in the cytoplasmic membrane; thus, the membrane is destroyed, inducing pyroptosis.^6^ IHC results showed that cleaved GSDMD protein was almost non-expressed in kidney tissue of the control group but was overexpressed in the CHn group. The active cleaved GSDMD protein was expressed merely in the epithelial cell membrane of renal tubules but not in glomerular and interstitial tissues (figure 2B). This supports that patients with CHn had GSDMD-executed pyroptosis in tubular epithelial cells, and pyroptosis played a part in hydronephrosis progression. Caspase-4 protein was expressed in renal tubular cells and showed overexpression in patients with CHn (figure 2C). Caspase-1 p20 protein (figure 2D) and activity NLRP3 protein (figure 2E) were widely expressed in renal tissue, but there was no significant difference between the two groups.

In summarizing the clinical results, the kidney tissue with CHn underwent severe renal interstitial fibrosis. We analyzed the causes of renal interstitial fibrosis in these children, presumably because LPS in the cell wall of Gram-negative bacilli activated caspase-4/GSDMD non-typical pyroptosis in the renal tubular epithelium, leading to pyroptosis in the tubular epithelium and thus promoting renal interstitial inflammation, which exacerbated fibrosis. It is also possible that LPS inhibited the expression of SIRT1 in renal tubular epithelial cells and reduced the protective effect of SIRT1.

### LPS can inhibit the proliferation of HK-2 cells and promote the messenger RNA expression of pyroprotein in human tubular epithelial cells

**Figure 3:**
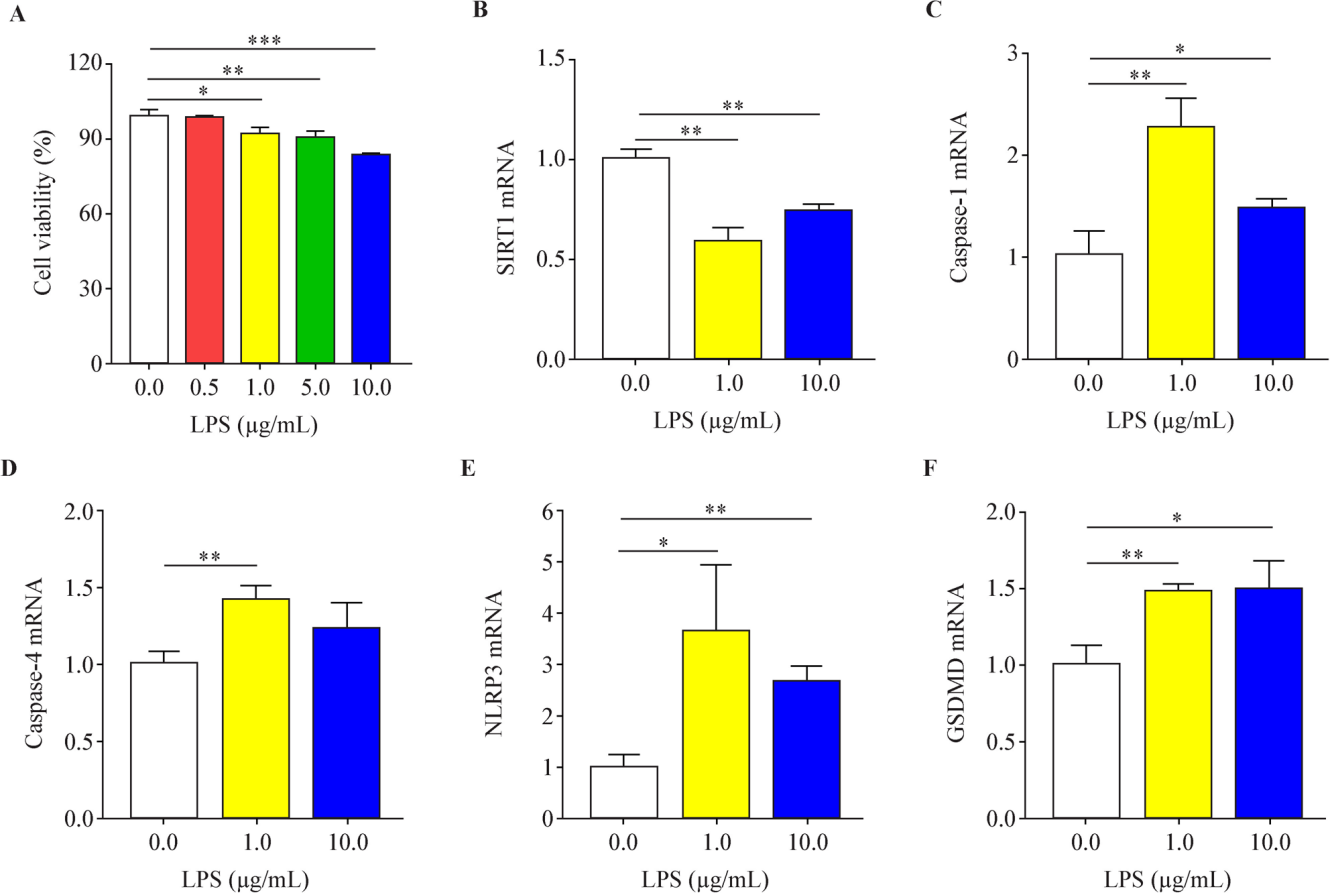
Effects of different concentrations of lipopolysaccharide (LPS) on the messenger RNA (mRNA) expression of HK-2 cell viability and pyroptosis-related markers. (A) The effect of different concentrations of LPS on the proliferation of HK-2 cells, higher concentration of LPS made the inhibition of cell activity more effective. (B) LPS inhibited the mRNA expression of sirtuin 1 (SIRT1) in HK-2 cells. (C) LPS promoted the mRNA expression of caspase-1 in HK-2 cells. (D) LPS promoted the mRNA expression of caspase-4 in HK-0 cells. (E) LPS promoted the mRNA expression of NLRP3 in HK-2 cells. (F) LPS promoted mRNA expression of gasdermin D (GSDMD) in HK-2 cells (*p<0.05, **p<0.01, ***p<0.001, compared with the 0.0 μg/mL LPS group, n=3).

IHC showed that pyroptosis mostly occurred in renal tubular epithelial cells; thus, HK-2 cells were cultured in vitro to build the model. The cells were treated with different concentrations of LPS (0.0, 0.5, 1.0, 5.0, 10.0 µg/mL) for 48 hours before cell proliferation was detected by a CCK8 kit. As shown in figure 3A, a high concentration of LPS inhibited the proliferation of HK-2 cells, and the inhibition became more effective as the concentration increased (figure 3A).

qRT-PCR indicated that the final concentrations (1.0 µg/mL and 10.0 µg/mL) of LPS inhibited the mRNA expression of SIRT1 (figure 3B) and promoted the mRNA expression of caspase-1 (figure 3C), caspase-4 (figure 3D), NLRP3 (figure 3E), and GSDMD (figure 3F) in HK-2 cells. Based on previous literature, we finally chose 1.0 µg/mL LPS for cell intervention in the follow-up experiments. The results demonstrated that LPS can activate HK-2 cells and cause pyroptosis, which is likely to be associated with the inhibition of SIRT1, which is identical to the results of clinical trials.

### Effect of SIRT1 on the expression of pyroptosis pathway proteins in HK-2 cells

**Figure 4:**
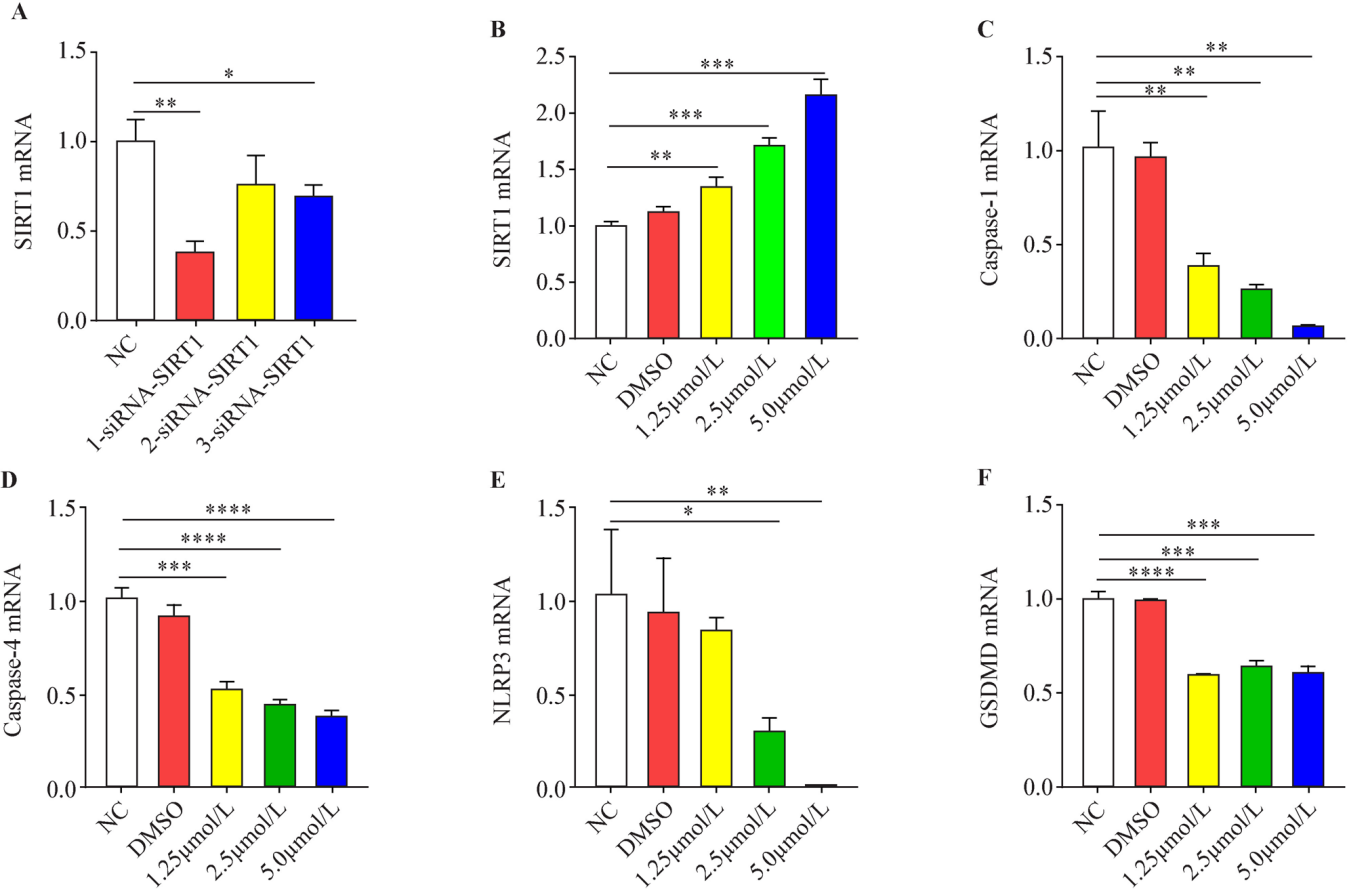
Effects of sirtuin 1 (SIRT1) on the messenger RNA (mRNA) expression of pyroptosis-associated protein in HK-2 cells. (A) The small interfering RNA (siRNA)-SIRT1 sequence with the optimum inhibitory effect was screened to find the most noticeable inhibitory effect of the first siRNA sequence. (B) SRT1720 could evidently promote the expression of SIRT1 mRNA in HK-2 cells, higher concentration made contributions to greater promoting effect. (C) SRT1720 inhibited the expression of mRNA of caspase-1 in HK-2 cells, the inhibitory effect became enhanced as the concentration increased. (D) SRT1720 inhibited the expression of mRNA of caspase-4 in HK-2 cells, the inhibitory effect became enhanced as the concentration increased. (E) SRT1720 inhibited the expression of mRNA of NLRP3 in HK-2 cells, the inhibitory effect became enhanced as the concentration increased. (F) SRT1720 inhibited the expression of mRNA of gasdermin D (GSDMD) in HK-2 cells, the inhibitory effect became enhanced as the concentration increased (*p<0.05, **p<0.01, ***p<0.001, ****p<0.0001 vs NC group, n=3). DMSO, dimethylsulfoxide.

Three siRNA-SIRT1 sequences were constructed and transfected into HK-2 cells for 48 hours. Afterwards, the expression of SIRT1 mRNA in HK-2 cells was measured by qRT-PCR. The best inhibitory sequence was selected for subsequent experiments (figure 4A). SRT1720, a specific agonist, was administered to HK-2 cells for 48 hours at different final concentrations of 0.0, 1.25, 2.5 and 5.0 µmol/L. Then, the mRNA expression of SIRT1 and pyroptosis-associated protein in HK-2 cells was measured by qRT-PCR. It was found that different concentrations of SRT1720 could promote the mRNA expression of SIRT1 in HK-2 cells, and higher concentrations could enhance the promoting effect (figure 4B). SRT1720 inhibited the mRNA expression of caspase-1 (figure 4C), caspase-4 (figure 4D), NLRP3 (figure 4E), and GSDMD (figure 4F) in HK-2 cells, showing a dose-dependent inhibitory effect, which proves that overexpression of SIRT1 can significantly inhibit the mRNA expression of pyroptosis-associated proteins in HK-2 cells.

### Effects of SIRT1 on the proliferative ability of HK-2 cells and the expression of IL-1β and IL-18 in the cell supernatant

**Figure 5:**
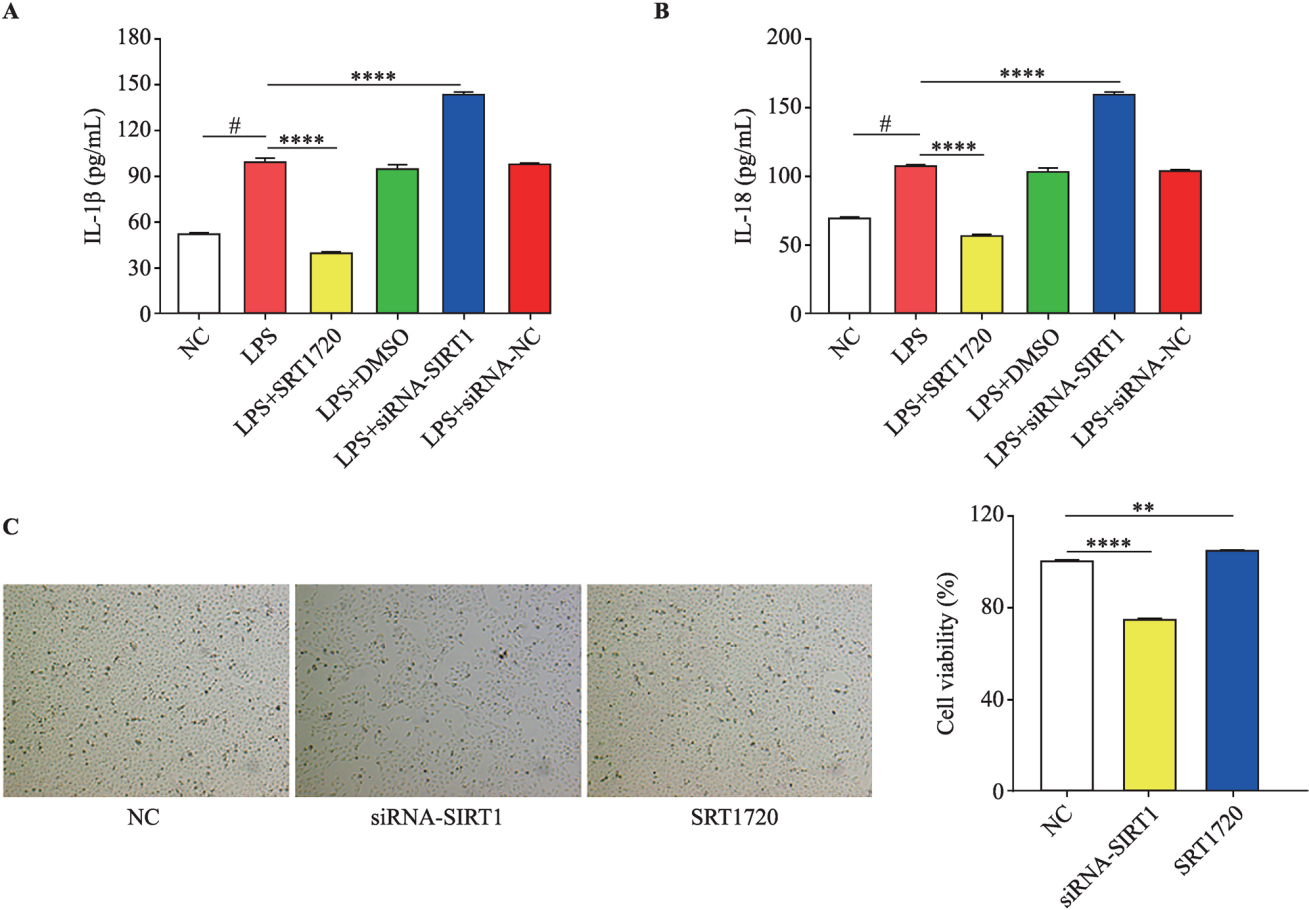
Effects of sirtuin 1 (SIRT1) on proliferative ability of HK-2 cells and he expression of interleukin (IL)-1β and IL-18 in cell supernatant. (A) Effects of lipopolysaccharide (LPS) and SRT1720 on IL-1β release in HK-2 cells: LPS promoted IL-1β release, SRT1720 inhibited IL-1β release, and small interfering RNA (siRNA) promoted IL-1β release. (B) Effects of LPS and SRT1720 on IL18 release in HK-2 cells: LPS promoted IL-18 release, SRT1720 inhibited IL-18 release, and siRNA promoted IL-18 release. (C) Effects of SIRT1 on HK-2 cell proliferation: SRT1720 promoted cell proliferation, and siRNA inhibited SIRT1 expression significantly inhibited the proliferation (**p<0.01, ****p<0.0001 vs NC group, n=3. #p<0.05, LPS group was compared with NC group, n=3).

ELISA results showed that LPS could promote the expression of IL-1β and IL-18 in the cell supernatant. siRNA promoted the expression of IL-1β and IL-18 in the cell supernatant by inhibiting SIRT1 expression, and SRT1720 inhibited the expression of IL-1β (figure 5A) and IL-18 (figure 5B) by promoting SIRT1 expression. SRT1720 protected the proliferative ability of HK-2 cells by promoting SIRT1 expression (figure 5C).

### Effects of SIRT1 on non-canonical pyroptosis pathway in HK-2 cells

**Figure 6:**
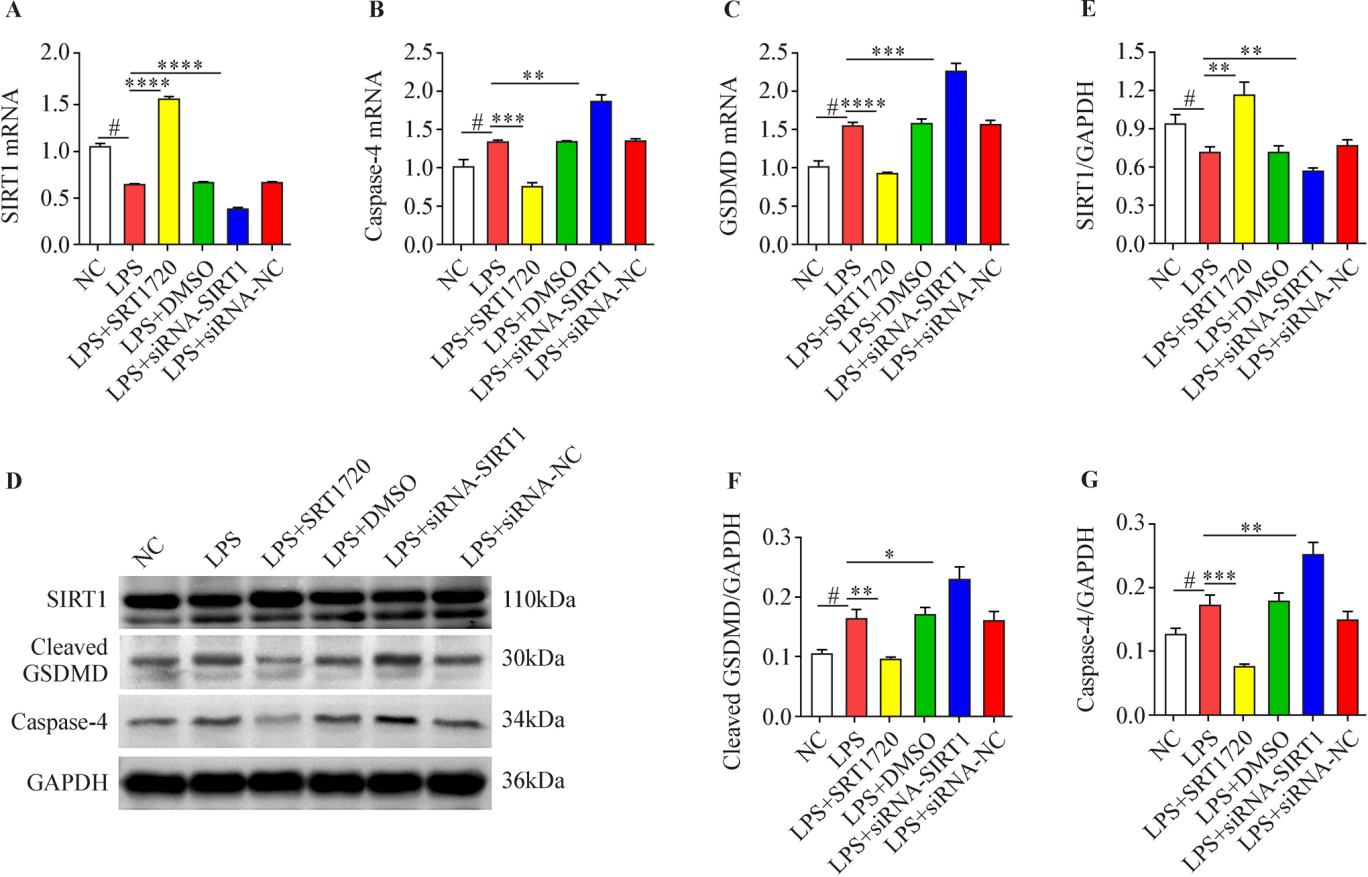
Sirtuin 1 (SIRT1) inhibits the development of non-canonical pyroptosis pathway in HK-2 cells. (A) Effects of SRT1720 and small interfering RNA (siRNA)-SIRT1 on the mRNA expression of SIRT1 in HK-2 cells in the environment with lipopolysaccharide (LPS). (B) Effects of SRT1720 and siRNA-SIRT1 on mRNA expression of caspase-4 in HK-2 cells. (C) Effects of SRT1720 and siRNA-SIRT1 on mRNA expression of gasdermin D (GSDMD) in HK-2 cells. (D, E, F, G) Effects of SRT1720 and siRNA-SIRT1 on protein expression of SIRT1, cleaved GSDMD, and caspase-4 in HK-2 cells (#p<0.05, LPS group vs NC group; *p<0.05, **p<0.01, ***p<0.001, ****p<0.0001 vs NC group, n=3).

LPS (1.0 µg/mL) inhibited the expression of SIRT1 in HK-2 cells, and LPS promoted the expression of caspase-4 and cleaved GSDMD in HK-2 cells. In the LPS (1.0 µg/mL) environment, we promoted and inhibited the expression of SIRT1 in HK-2 cells. The results showed that SRT1720 promoted SIRT1 expression in HK-2 cells and inhibited caspase-4 and cleaved GSDMD expression. siRNA inhibition of SIRT1 expression further increased the effect of LPS (figure 6).

### Effects of SIRT1 on the canonical pyroptosis pathway in HK-2 cells

**Figure 7:**
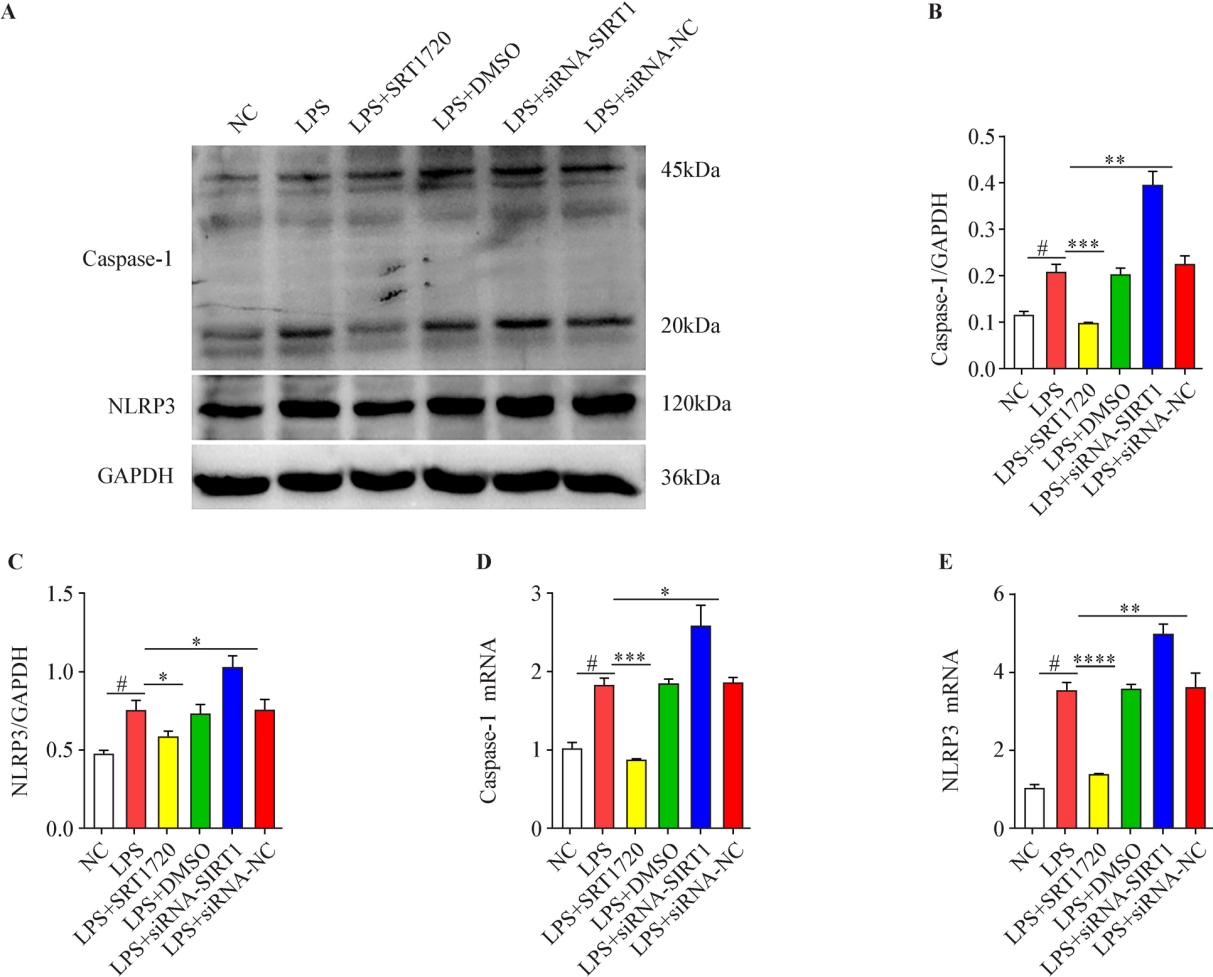
Sirtuin 1 (SIRT1) inhibits the development of canonical pyroptosis pathway in HK-2 cells. (A, B, C) The effects of SRT1720 and small interfering RNA (siRNA)-SIRT1 on protein expression of caspase-1 and NLRP3 in HK-2 cells in the environment with lipopolysaccharide (LPS). (D) Effects of SRT1720 and siRNA-SIRT1 on mRNA expression of caspase-1 in HK-2 cells. (E) Effects of SRT1720 and siRNA-SIRT1 on mRNA expression of NLRP3 in HK-2 cells (#p<0.05, LPS group vs NC group; *p<0.05, **p<0.01, ***p<0.001, ****p<0.0001 vs NC group, n=3). DMSO, dimethylsulfoxide.

qRT-PCR and western blot analysis showed that LPS could promote the mRNA and protein expression of caspase-1 and NLRP-3 in HK-2 cells. SRT1720 inhibited the mRNA and protein expression of caspase-1 and NLRP3 in the cells. Inhibition of SIRT1 expression further enhanced the promoting effect of LPS (figure 7).

Through in vitro cytology experiments, it was concluded that overexpression of SIRT1 is favorable to the proliferation of HK-2 cells, reduces the incidence of pyroptosis and the release of the inflammatory factors IL-1β and IL-18, and then alleviates the inflammatory reaction to protect the kidney. LPS can activate pyroptosis in HK-2 cells and increase the degree of inflammatory injury, while SIRT1 may be the target of LPS, and its overexpression can reverse LPS-activated pyroptosis. In combination with the results of clinical experiments, the following conclusion can be drawn: overexpression of SIRT1 can reduce the release of the inflammatory factors IL-1β and IL-18 by inhibiting the non-typical LPS-activated pyroptosis pathway, protect renal tubular epithelial cells from death, and thus slow the progression of renal fibrosis.

## Discussion

Recently, much has been learned about the pathophysiology of obstructive nephropathy and many novel biomarkers have been investigated for diagnostic and prognostic purposes. Some biomarkers offer promising results, such as TGF-β1, epidermal growth factor, kidney injury molecule 1.^17^ LPS can promote the progression of renal interstitial fibrosis in unilateral ureteral occlusion (UUO) by activating the TGF-β1 signaling pathway. In this retrospective study, 13 patients with CHn had urinary tract infections before surgery. The major pathogenic bacteria that cause urinary tract infections are Gram-negative bacteria. It has been demonstrated that LPS in the cell wall of Gram-negative bacteria can promote inflammatory injury and renal interstitial fibrosis in the renal tissues of rats with obstructive hydronephrosis (UUO).^1^ The serum levels of hs-CRP, PCT, IL-6, and IFN-γ in these children with CHn were elevated to varying degrees during urinary tract infection. IL-6 and IFN-γ have been shown to promote renal fibrosis.^18^ Masson staining showed that the patients with CHn had advanced to severe renal interstitial fibrosis at the end stage. Conclusion These clinical trial data confirm that LPS is an important factor promoting the progression of renal interstitial fibrosis in CHn.

Relieving inflammatory reactions and tissue damage is an important method to address renal fibrosis. Studies have shown that increasing the expression of SIRT1 inhibits inflammatory reactions and reduces fibrosis in a variety of kidney diseases.^19^ LPS can promote inflammation by inhibiting SIRT1 expression in kidney tissue.^20^ Specific agonists of SIRT1 can antagonize LPS, reduce the release of inflammatory factors, alleviate tissue damage, and slow fibrosis progression.^21^ In this study, IHC results showed that the expression of SIRT1 protein decreased in CHN-affected kidney tissue, and in vitro cell experiments showed that LPS inhibited SIRT1 expression in HK-2 cells, and the SIRT1-specific agonist SRT1720 could antagonize the inhibitory effect of LPS. Therefore, we speculate that the mechanism of LPS promoting the progression of renal interstitial fibrosis in CHn may be that LPS inhibits SIRT1 expression in renal tubular epithelial cells, leading to a decline in the ability of SIRT1 to inhibit inflammation and thus promoting the progression of renal interstitial fibrosis.

The active GSDMD-N-terminus can bind to the lipid components on the cell membrane to form 10–15 nm pores through which mature IL-1β and IL-18 can pass.^7^ It was reported that necrosulfonamide (NSA), an inhibitor of GSDMD-N, inhibited LPS-activated pyroptosis and reduced the inflammatory reaction.^22^ The results of this study showed that the expression of GSDMd-N-terminal protein was increased in CHN-affected kidney tissues and was mainly expressed in tubular epithelial cells, while there was almost no positive expression in control kidney tissues. At the same time, the expression of caspase-4 protein in patients with CHn was increased. The cell assay results showed that LPS promoted the expression of caspase-4 in HK-2 cells, cleaved caspase-1, NLRP3, and cleaved GSDMD, promoted the release of IL-1β and IL-18 in HK-2 cells, and induced cytokinesia. These results suggest that LPS-activated caspase-4/GSDMD non-canonical pyroptosis may be involved in promoting the progression of renal interstitial fibrosis in CHn. The mechanism may be that LPS directly recognizes and binds caspase-4 in renal tubular epithelial cells. After binding, caspase-4 becomes oligomeric and activated and then cleaves GSDMD to form the GSDMd-N end, inducing cell scorching, releasing IL-1β and IL-18 to trigger an inflammatory cascade, and then promoting the progression of renal interstitial fibrosis.^23^ Studies have confirmed that pyroptosis can promote the progression of fibrosis in heart and liver tissues. The mechanism may be that pyroptosis promotes inflammation and activates the profibrosis signaling pathway.^24 25^ Therefore, LPS is an important mechanism that promotes pyroptosis in renal interstitial fibrosis in CHn by promoting pyroptosis.

Recent studies show that the agonist melatonin of SIRT1 can inhibit the expression of the LPS-promoted NLRP3 inflammasome and reduce the release of IL-1β.^26^ Overexpression of SIRT1 inhibits pyroptosis of liver cells and reduces inflammatory reactions and cell injury in ischemic stress mice.^27^ SIRT1 agonists can inhibit the activation of the NLRP3 inflammasome in cardiomyocytes.^28^ Gardeniasin can activate the expression of SIRT1 to reduce the incidence of NLRP3/cleaved caspase-1 and GSDMD-N-mediated pyroptosis and alleviate inflammatory reactions and injury in renal tissue.^29^ SIRT1 can reduce the incidence of pyroptosis in human tubular epithelial cells with the help of deacetylation.^30^ The results are consistent with our findings that overexpression of SIRT1 inhibited pyroptosis of HK-2 cells and reduced the release of the inflammatory factors IL-18 and IL-1β. At the same time, we found that promoting SIRT1 expression could inhibit the NLRP3/caspase-1 classical cell pyrodeath pathway and inhibit the caspase-4 non-classical cell pyrodeath pathway, which is our new discovery. Therefore, we speculate that promoting SIRT1 expression in CHN-affected renal tissues can reduce inflammation by inhibiting pyroptosis, which may be an important mechanism by which SIRT1 alleviates renal interstitial fibrosis in children with CHn. In addition, excessive death of renal tubule epithelial cells caused by cell pyrosis is also one of the factors promoting the progression of renal interstitial fibrosis. SRT1720 can protect the proliferation ability of renal tubular epithelial cells by promoting SIRT1 expression, and maintaining cell proliferation ability is also a possible mechanism by which SIRT1 improves renal fibrosis.

It was found that nuclear factor kappa B (NF-κB) p65 could promote the expression of typical pyroptosis activated by NLRP3/cleaved caspase-1.^31^ More reactive oxygen species (ROS) in vivo could promote the expression of caspase-1 in cells, induce pyroptosis, and help release inflammatory factors.^32^ Previous studies have demonstrated that deacetylation of SIRT1 can inhibit the expression and activity of NF-κB p65 and ROS, inhibit the release of inflammatory factors, and relieve inflammatory injury in kidney tissues.^33^ Combined with the results of this study, we hypothesized that SIRT1 is an important target in the pyrodeath process of LPS-activated cells and that promoting SIRT1 expression can inhibit the pyrodeath of LPS-activated cells by inhibiting NF-κB p65 and ROS.

The LPS-activated caspase-4 non-classical pyroptosis pathway may play an important role in clinical experiments, which was also confirmed by cytological results. The results of clinical experiments showed no significant difference in NLRP3/caspase-1 classic pyroptosis pathway expression, and cytological experiments showed that LPS could also promote the expression of NLRP3 and caspase-1. The possible reason for this phenomenon was that after LPS activated caspase-4 non-classical pyroptosis pathway, activated caspase-4 could activate the NLRP3 inflammasome, thus promoting caspase-1 activation, accelerating IL-18 and IL-1β maturation, and inducing pyroptosis.^34^ This may also be due to the small number of experimental specimens leading to statistical bias. Alternatively, the paracancerous tissue of Wilms tumors cannot completely replace normal kidney tissue because the current research results suggest that activating pyrogenesis can inhibit the proliferation and migration of malignant tumor cells, and pyrogenesis has the potential to treat malignant tumors.^35^

In conclusion, LPS can inhibit SIRT1 expression and promote the occurrence of caspase-4/GSDMD non-classical cell scorching in renal tubular epithelial cells when urinary tract infection caused by Gram-negative bacteria occurs in children with CHn, thus promoting the progression of renal interstitial fibrosis. SIRT1 is an important target of LPS-activated pyrodeath cells. SRT1720 can promote SIRT1 to inhibit LPS-activated pyrodeath cells, reduce the release of IL-18 and IL-1β, and alleviate the progression of renal interstitial fibrosis. This conclusion is innovative to some extent, which is a new explanation of the progression mechanism of renal interstitial fibrosis in children with CHn and provides a new idea for the comprehensive treatment of children with CHn. The SIRT1-specific agonist SRT1720 has the potential to improve renal fibrosis and salvage renal function in children with CHn.

In addition, this study also found that SIRT1 inhibited the NLRP3/caspase-1-mediated classical pyrodeath pathway and inhibited non-classical pyrodeath pathway mediated by caspase-4. This is also an innovative finding of this study. Although the results of this study are encouraging, there are still many deficiencies in the experimental design, such as the small number of clinical specimens and the lack of relevant animal experiments. In the future, prospective studies are needed to evaluate the clinical usefulness of urinary biomarkers in the diagnosis and follow-up of children with obstructive nephropathy. We detected the expression levels of IL-1β and IL-18 in amniotic fluid and the urine of newborn hydronephros. We will further confirm pyroptosis as a potential therapeutic target in animal models of CHn.

## Data Availability

All data relevant to the study are included in the article or uploaded as supplementary information.
