# Supplementary material for: Role and significance of SIRT1 in regulating the LPS-activated pyroptosis pathway in children with congenital hydronephrosis
Source: World J Pediatr Surg. 2023 Jul 31;6(4):e000602. doi: 10.1136/wjps-2023-000602 (PMC10391807; doi:10.1136/wjps-2023-000602)
Supplement: Supplementary data [file wjps-2023-000602supp001.pdf]

Table 1 The three gene sequences of siRNA-SIRT1

Gene name	Sense (5'-3')	Antisense (5'-3')
1-siRNA-SIRT1	CGGUGAUGAAAUUAUCACUAA TT	UUAGUGAUAAUUUCAUCACCG TT
2-siRNA-SIRT1	CAUGAAGUGCCUCAGAUAUUA TT	UAAUAUCUGAGGCACUUCAUG TT
3-siRNA-SIRT1	GCGGCUUGAUGGUAAUCAGUA TT	UACUGAUUACCAUCAAGCCGC TT

Table 2 Gene sequences of each target primer

Primer name		Sense (5'-3')	Size (bp)
h-Caspase-1	F	TAAGAAAGCCCACATAGAGAAGGAT	105
	R	AGTCTTCCAATAAAAACAGAGCCC	
h-Caspase-4	F	CAATAAAGGAGAGAAACAACCGC	219
	R	AGGACTTGTGCTCTGGTCTGGTAG	
h-NLRP3	F	AGCACTAATCAGAATCTCACGCACC	180
	R	GCTGGAGGTCAGAAGTGTGGAAAG	
h-GSDMD	F	GGACCCTAACACCTGGCAGACTC	146
	R	GCGTGACTTCCACCTCCTTCTG	
h-GAPDH	F	GAAGGTGAAGGTCGGAGTC	227
	R	GAAGATGGTGATGGGATTTC	
h-SIRT1	F	GGCAAAGGAGCAGATTAGTAGGC	180
	R	GGTTCTTCTAAACTTGGACTCTGGC	
